# Triptolide inhibits human immunodeficiency virus type 1 replication by promoting proteasomal degradation of Tat protein

**DOI:** 10.1186/s12977-014-0088-6

**Published:** 2014-10-17

**Authors:** Zhitao Wan, Xulin Chen

**Affiliations:** State Key Laboratory of Virology, Wuhan Institute of Virology, Chinese Academy of Sciences, Wuhan, Hubei People’s Republic of China; Current address: China Novartis Institutes for BioMedical Research, Shanghai, People’s Republic of China

**Keywords:** HIV-1, Triptolide, Antiviral, Tat, Proteasomal degradation

## Abstract

**Background:**

Plants remain an important source of new drugs, new leads and new chemical entities. Triptolide is a diterpenoid epoxide isolated from *Tripterygium wilfordii* Hook F that possesses a broad range of bioactivities, including anti-inflammatory, immunosuppressive and anti-tumor properties. The antiviral activity of triptolide against human immunodeficiency virus type 1 (HIV-1) has not been reported.

**Results:**

In this study, nanomolar concentrations of triptolide were shown to potently inhibit HIV-1 replication *in vitro*. To identify the step(s) of the HIV-1 replication cycle affected by triptolide, time-of-addition studies, PCR analysis and direct transfection of viral genomic DNA were performed. The results of these experiments indicated that triptolide acts at the stage of viral gene transcription. In addition, a luciferase-based reporter assay that allows quantitative analysis of long terminal repeat (LTR)-driven transcription showed that Tat-induced LTR activation was impaired in the presence of triptolide. Moreover, Western blot analysis of exogenous gene expression (driven by the human elongation factor 1 α subunit promoter) in transiently transfected cells revealed that triptolide specifically reduces the steady-state level of Tat protein, without suppressing global gene expression. Further studies showed that triptolide accelerates Tat protein degradation, which can be rescued by administration of the proteasome inhibitor MG132. Mutation analysis revealed that N-terminal domains of Tat protein and nuclear localization are required for triptolide to reduce steady-state level of Tat.

**Conclusion:**

This study suggests for the first time that triptolide exerts its anti-HIV-1 activity by specifically prompting the degradation of the virally encoded Tat protein, which is a novel mechanism of action for an anti-HIV-1 compound. This compound may serve as a starting point for developing a novel HIV-1 therapeutic approach or as a basic research tool for interrogating events during viral replication.

## Background

The current therapeutic strategy for the treatment of human immunodeficiency virus type 1 (HIV-1) infection and acquired immune deficiency syndrome (AIDS) is highly active antiretroviral therapy (HAART) [1]. Although HAART have been shown to be successful in controlling viral replication and disease progression, issues of multidrug resistance, side effects and poor compliance continue to raise concerns [2-4]. Furthermore, given the presence of long-lived latently infected cells, the current HAART formula does not eradicate the virus even after a prolonged period of treatment [5]. Thus, discovery and development of novel therapies for the treatment of HIV-1 infection is still needed.

HIV-1 gene transcription is an essential step in the viral life cycle and the only stage during which viral genome amplification occurs. HIV-1 transcription is predominantly directed by a promoter in the 5′ long terminal repeat (5′ LTR) of the integrated provirus and is regulated by viral regulatory proteins as well as cellular factors [6]. The virally encoded Tat protein is essential for efficient transcription and plays a central role in sustaining a high level viral replication [7,8]. Tat protein directly binds to the trans-activating response element (TAR), an RNA stem-loop structure located at the 5′ ends of nascent HIV-1 transcripts. In binding to TAR, Tat recruits a positive transcription elongation complex (P-TEFb) composed of cyclin T1 (CycT1) and cyclin-dependent kinase 9 (CDK9) [9,10]. Subsequently, CDK9 phosphorylates the C-terminal domain of RNA polymerase II and thus enhances transcription elongation [11]. The transcriptional machinery, which depends upon the intricate interplay between the viral regulatory protein Tat and various host components, represents a potential therapeutic target that has not been exploited by currently available antiretroviral drugs [12]. Up to this point, intensive research efforts have focused on the discovery and development of selective HIV-1 replication inhibitors targeting Tat-mediated transcription. These inhibitors include the Tat/TAR/ P-TEFb interaction inhibitors and molecules that interfere with host factors [13-22]. However, none of these inhibitors have advanced to clinical trials because of their potential toxicity, despite extensive research.

*Tripterygium wilfordii* Hook F is a Chinese herd with an established history of use in the treatment of rheumatoid arthritis. Triptolide is a diterpenoid epoxide isolated from *Tripterygium wilfordii* Hook F with a broad range of bioactivities, including anti-inflammatory, immunosuppressive and anti-tumor properties [23]. In this study, triptolide was found to potently inhibit HIV-1 replication at nanomolar concentrations. In a manner distinct from all previously described HIV-1 transcription inhibitors, mode-of-action studies have demonstrated that triptolide specifically enhances Tat protein degradation, resulting in suppression of LTR-mediated viral gene transcription.

## Results

### Triptolide inhibits HIV-1 replication in vitro

**Figure 1:**
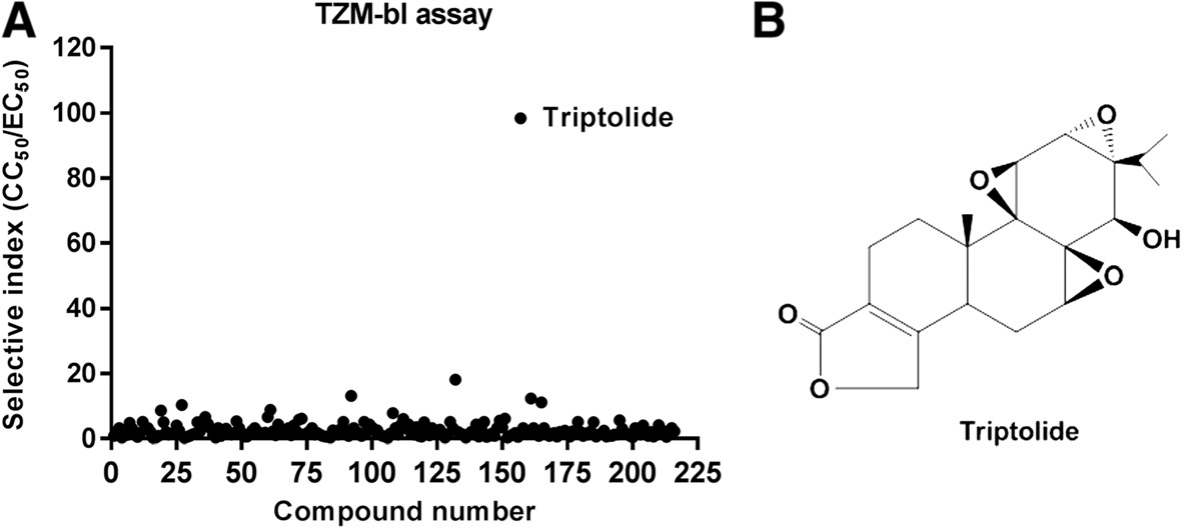
**Screen of natural compounds led to the identification of triptolide as a novel anti-HIV-1 agent. (A)** TZM-bl cells were infected with HIV-1 NL4.3 at an MOI of 0.5 in the presence of serially diluted test compounds. Virus replication was quantified by measuring luciferase expression at 48 h post-infection. The cytotoxicity of the tested compounds was evaluated in parallel with their antiviral assays. The anti-HIV-1 activity of each compound was presented as selective index, the ratio of CC_50_ to EC_50_. **(B)** Chemical structure of triptolide.

In an effort to identify novel anti-HIV-1 inhibitors, more than 200 highly purified natural compounds were screened using TZM-bl cells and replication competent HIV-1 (NL4-3 strain). TZM-bl cells are permissive to HIV-1 infection and harbor an integrated copy of the luciferase gene under transcriptional control of the HIV-1 5′ LTR promoter. Reporter gene expression is induced by viral Tat protein upon infection. Thus, compounds targeting any stage from viral entry to viral gene expression can be detected in this assay. The cytotoxicity of the tested compounds was also evaluated in parallel with the antiviral assay. For each compound, the concentration which inhibits luciferase expression by 50% (EC_50_) and the concentration which reduces cell viability by 50% (CC_50_) were calculated using logistic regression analysis. As shown in Figure 1A and B, among all the compounds screened, triptolide showed the highest selective index (SI, the ratio of CC_50_ to EC_50_). Following primary screening, the antiviral activity of triptolide was evaluated in a panel of *in vitro* cell-based assays.

**Figure 2:**
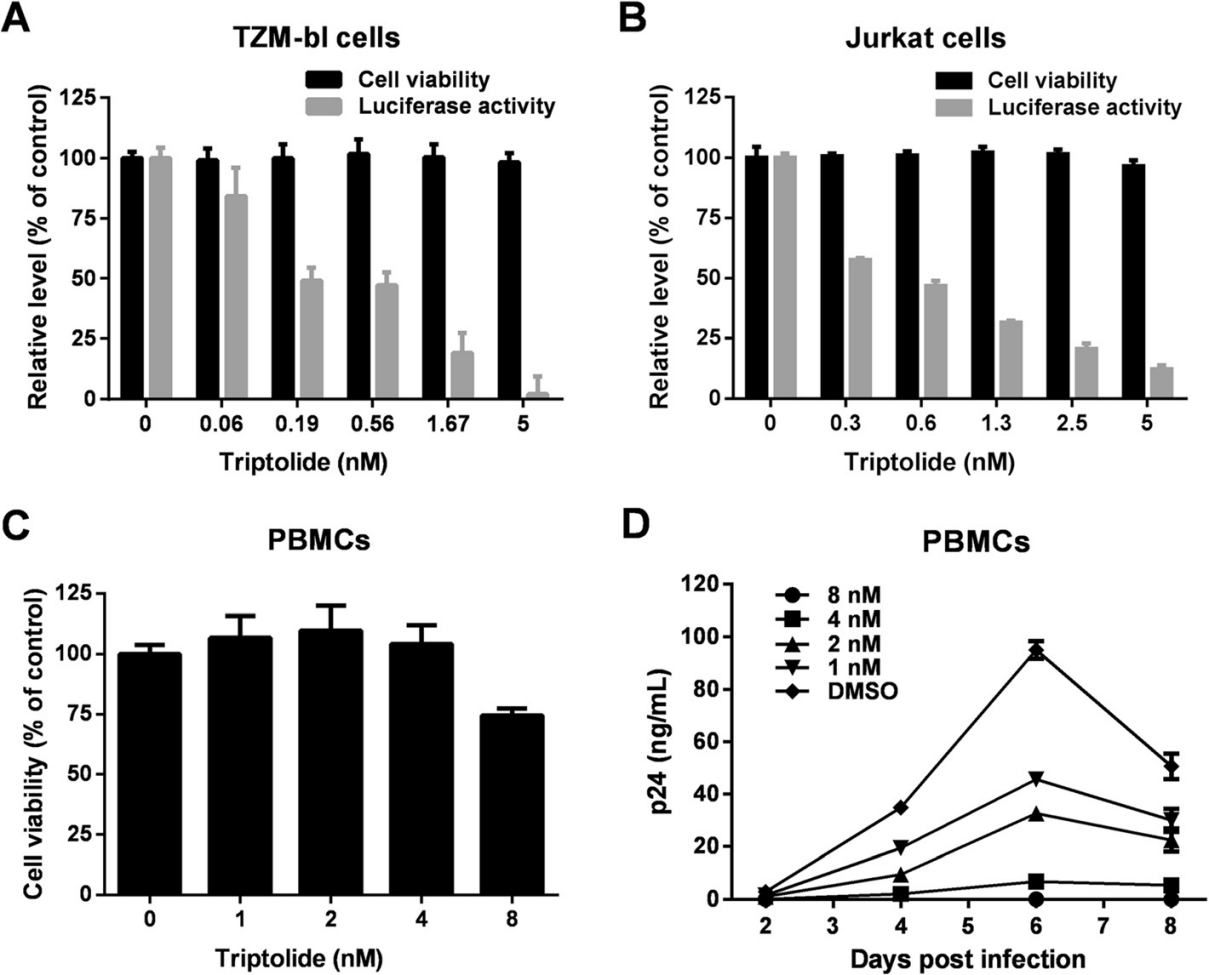
**Triptolide potently inhibits HIV-1 replication** ***in vitro*** **. (A)** The effect of triptolide on HIV-1 replication in TZM-bl reporter cells. TZM-bl cells were infected with HIV-1 (MOI = 0.5, strain NL4-3) in the presence of 3-fold serial dilutions of triptolide. Virus replication was quantified by measuring luciferase expression at 48 h post-infection. A cell viability assay was carried out in parallel using CellTiter-Glo. **(B)** The effect of triptolide on HIV-1 reporter virus replication in Jurkat cells. Jurkat cells were infected with HIV-1-Luc (pNL4-3.Luc.R-E- pseudotyped with the NL4-3 envelope) at an MOI of 0.5 in the presence of triptolide. Luciferase gene expression was quantified at 48 h post-infection. Compound cytotoxicity was determined in mock-infected cells. **(C)** Cytotoxicity of triptolide in PBMCs. PBMCs from donor A were treated with the indicated concentrations of triptolide for six days before cell viability examination using CellTiter-Glo. **(D)** Inhibition of HIV-1 replication in PBMCs. PBMCs from donor A were infected with HIV-1 (MOI = 0.01, strain NL4-3) in the presence of increasing concentrations of triptolide. Supernatants were collected at the indicated days post-infection and virus replication levels were determined using p24 ELISA. Results were presented as the mean plus the standard deviations (*n* = 3).

We first confirmed the antiviral activity of triptolide in the TZM-bl assay. As shown in Figure 2A, a significant and dose-dependent inhibitory effect on virus replication was observed at concentrations in the nanomolar range (EC_50_ = 0.32 nM). At 5 nM, the compound reduced luciferase expression by 97.9%. To exclude the possibility that the inhibitory effect was due to nonspecific cytotoxicity, cell viability assays were performed in parallel, and no obvious toxicity was observed at all tested concentrations (Figure 2A).

Considering that the TZM-bl assay monitors the stages of infection up to the integration of viral cDNA into the host cell chromosome and the expression of viral gene, we presume that this compound may act at any stage from viral entry to viral gene expression. Next, the antiviral activity of triptolide was validated in Jurkat T lymphocytes using a single-cycle HIV-1 reporter virus (replication defective pNL4-3.Luc.E-R- packaged with an HIV-1 NL4-3 envelope). Upon integration into the host chromosome, this recombinant virus expresses the firefly luciferase gene, and consequently, luciferase activity in infected cells correlates with the rate of viral replication. Thus, compounds targeting any stage from viral entry to viral gene expression should be active in this assay. Consistent with our observations in the TZM-bl assay, triptolide treatment inhibited virus replication in a dose-dependent manner, with an EC_50_ of 0.45 nM (Figure 2B).

To further characterize its anti-HIV-1 activity, triptolide was evaluated in the p24 assay using peripheral blood mononuclear cells (PBMCs) as host cells. As shown in Figure 2C and D, the level of HIV-1 NL4-3 replication was significantly reduced in the presence of triptolide as measured by p24 production. At the maximum non-toxic concentration (4 nM), triptolide reduced p24 level by 89.4 to 99.1%, depending on the time of detection. Furthermore, triptolide also showed similar potency against two other HIV-1 strains, LAI and BaL, in PBMCs from different donors (See the summary of antiviral data in Table 1). Collectively, these results demonstrate that triptolide inhibits acute HIV-1 infection in various cell types.

**Table 1:** ***In vitro*** **anti-HIV-1 activity of triptolide**

**Virus**	**Cell type**	**Measurement**	**EC**_**50**_ **(nM)** ^***a***^	**CC**_**50**_ **(nM)** ^***b***^	**SI** ^***c***^
HIV-1 NL4-3	TZM-bl	Luciferase	0.32	25.4	79.4
HIV-1-Luc	Jurkat	Luciferase	0.45	13.9	30.9
HIV-1 NL4-3	PBMC donor A	p24	1.1^*d*^	12.6	11.5
HIV-1 NL4-3	PBMC donor B	p24	0.9^*d*^	15.3	17.0
HIV-1 NL4-3	PBMC donor C	p24	1.3^*d*^	11.2	8.6
HIV-1 LAI	PBMC donor A	p24	1.5^*d*^	12.6	8.4
HIV-1 LAI	PBMC donor B	p24	1.2^*d*^	15.3	12.8
HIV-1 LAI	PBMC donor C	p24	1.5^*d*^	11.2	7.5
HIV-1 BaL	PBMC donor A	p24	1.4^*d*^	12.6	9.0
HIV-1 BaL	PBMC donor B	p24	1.6^*d*^	15.3	9.6
HIV-1 BaL	PBMC donor C	p24	1.0^*d*^	11.2	11.2

^*a*^Compound concentration required to reduce the production of luciferase or p24 antigen by 50%.

^*b*^Compound concentration required to reduce mock-infected cell viability by 50%.

^*c*^Selective index, ratio of CC_50_/EC_50_.

^*d*^EC_50_ value was calculated based on p24 inhibition at day 6 post-infection.

### Triptolide inhibits HIV-1 gene transcription

**Figure 3:**
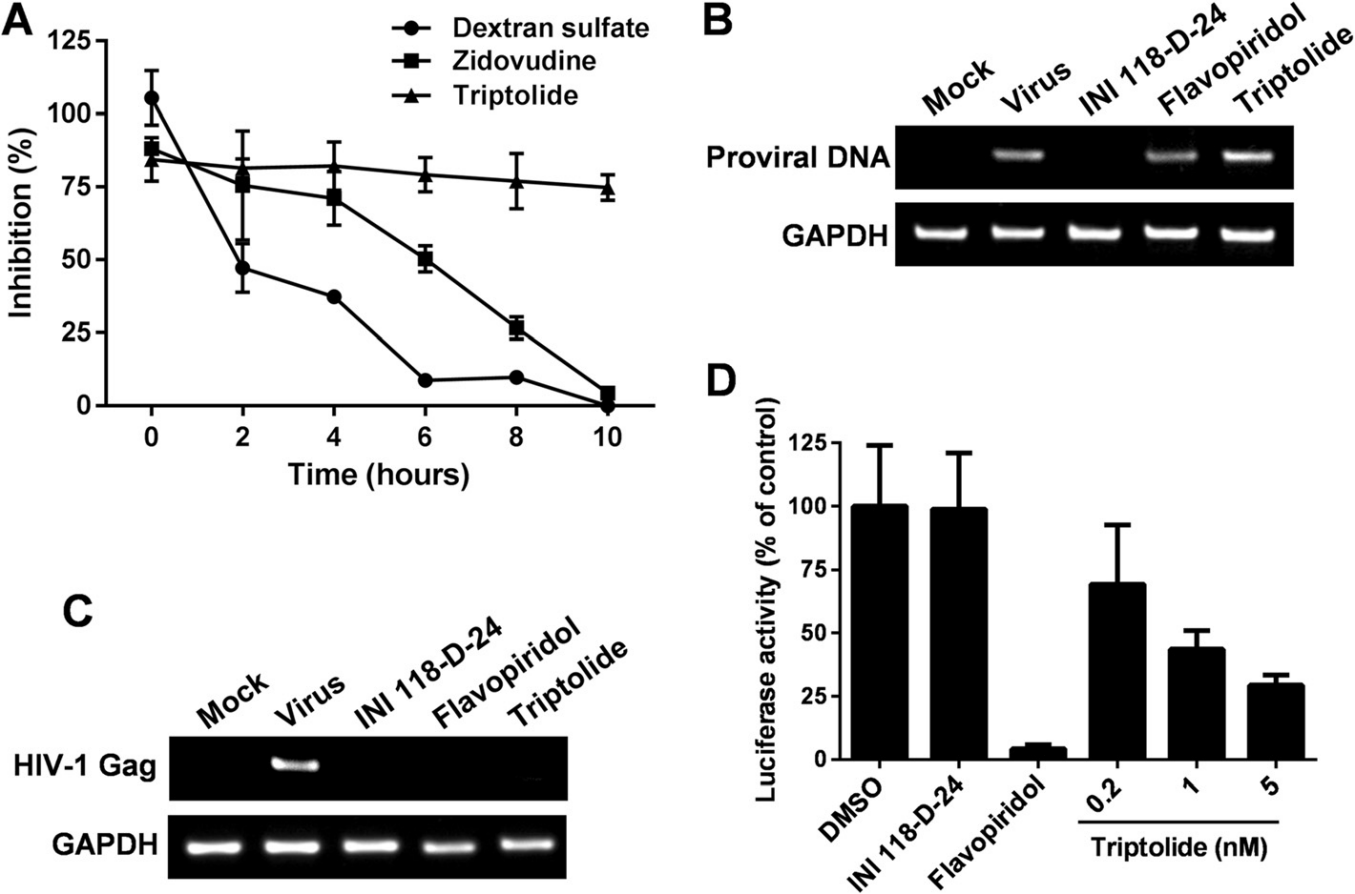
**Triptolide inhibits HIV-1 transcription. (A)** Time-of-addition analysis. TZM-bl cells were infected with HIV-1 (MOI = 0.5, strain NL4-3), and the virus inoculum was removed after 1 h of absorption. Test compounds (dextran sulfate at 10 μg/mL, zidovudine at 1 μM and triptolide at 5 nM) were added at the indicated time points after infection. Reporter signal was monitored at 48 h post-infection as a measure of viral replication. **(B)** The effect of triptolide on HIV-1 proviral DNA formation. Jurkat cells were infected with HIV-1 (MOI = 0.5, strain NL4-3) in the presence of the test compounds (INI 118-D-24, 50 μM; flavopiridol, 20 nM; and triptolide, 5 nM). Proviral DNA synthesis was determined by nested PCR analysis at 12 h post-infection. **(C)** The effect of triptolide on HIV-1 mRNA synthesis. Jurkat cells were infected with HIV-1(MOI = 0.5, strain NL4-3) in the presence of the test compounds (INI 118-D-24, 50 μM; flavopiridol, 20 nM; triptolide, 5 nM). Viral mRNA (the Gag region) content was determined by reverse-transcription PCR analysis 12 h post-infection. **(D)** Inhibition of luciferase expression encoded in pNL4-3.Luc.R-E- by tiptolide. HIV-1 molecular clone pNL4-3.Luc.R-E- and pRL-TK (transfection efficiency control) were co-transfected into Jurkat cells. Transfected cells were cultured in the presence of the test compounds (INI 118-D-24, 50 μM; flavopiridol, 20 nM; triptolide, 0.2 ~ 5 nM) for an additional 24 h before measuring luciferase activity with a dual-luciferase assay system. Results were presented as the mean plus the standard deviations (*n* = 3).

After confirmation of the anti-HIV-1 activity of triptolide, we sought to identify its mechanism of action. To pinpoint the stage targeted by triptolide during viral replication, time-of-addition experiment was performed. TZM-bl cells were infected with HIV-1 NL4-3, and triptolide and reference compounds were added to the infected cells at various time points. Viral replication level was quantified at 48 h post-infection by measuring luciferase activity. The inhibition profile of tirptolide was compared to the profiles of reference compounds with a known mechanism of action. As shown in Figure 3A, the viral entry inhibitor dextran sulfate showed a dramatic early loss in activity. However, the reverse-transcription inhibitor zidovudine retained significant inhibitory activity (71%) when added up to 4 h post-infection and lost its activity thereafter. In contrast, triptolide displayed a profile that was distinct from both references. Addition of triptolide could be postponed up to 10 h post-infection without losing activity, suggesting that this compound affects a stage that is post reverse-transcription.

The post-reverse-transcription events of the viral replication cycle include integration and gene expression. To elucidate the effect of triptolide on these two events, synthesis of proviral DNA and viral mRNA during single-round infection was examined by PCR analysis. Jurkat cells were infected with wild-type HIV-1 in the presence of triptolide and reference compounds, and total DNA and mRNA were extracted 12 h post-infection (the use of this early time point ruled out *de novo* infection) for the examination of integrated proviral DNA and viral mRNA. As shown in Figure 3B and C, the integrase inhibitor 118-D-24 [24] reduced proviral DNA formation and subsequent viral mRNA synthesis. However, triptolide along with the HIV-1 gene transcription inhibitor flavopiridol [16] had no effect on virus integration but did repress viral mRNA synthesis, suggesting that triptolide inhibits HIV-1 transcription from integrated proviral DNA.

To further confirm that triptolide interferes with HIV-1 transcription, a transient gene expression assay was performed. The HIV-1 molecular clone pNL4-3.Luc.E-R- was transfected into Jurkat cells in the presence of either triptolide or various reference compounds. At 24 h after transfection, viral gene transcription (indicated by luciferase activity) was determined. In this assay, the early events during viral replication, including entry, reverse transcription and integration were bypassed, and the direct effect of the test compounds on viral gene expression was examined. As expected, the integrase inhibitor 118-D-24 was inactive in the assay, and the gene expression inhibitor flavopiridol significantly reduced luciferase expression (Figure 3D). Interestingly, triptolide inhibited reporter expression in a dose-dependent manner with a potency comparable to levels observed in the antiviral assays, further suggesting that this compound acts at the stage of viral gene transcription.

### Triptolide inhibits Tat-mediated gene transcription

**Figure 4:**
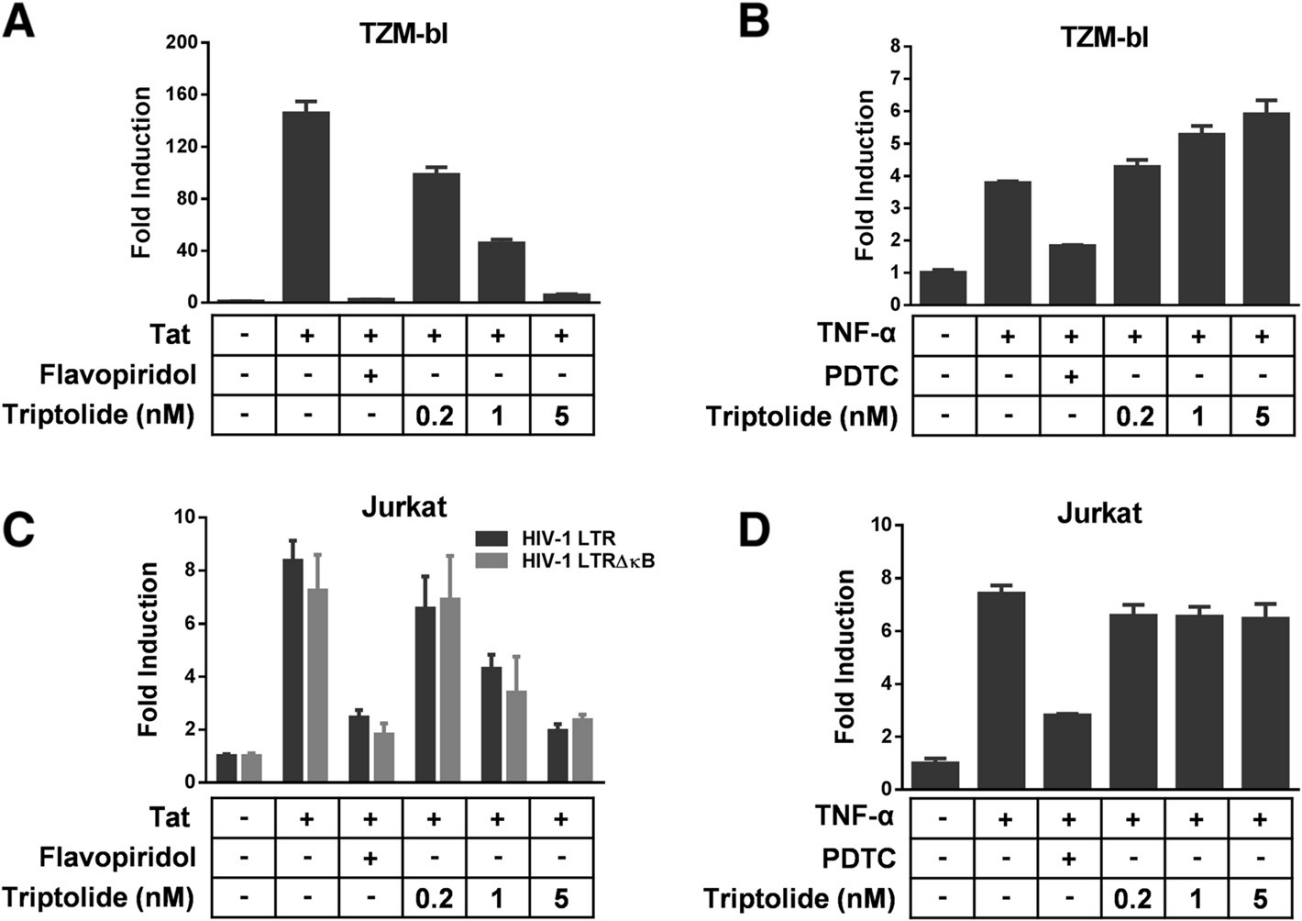
**Triptolide inhibits Tat-mediated LTR activation. (A)** TZM-bl cells were co-transfected with pEF1-Tat and pRL-TK, and treated with the test compounds. Luciferase activity was measured at 48 h post-transfection. **(B)**TZM-bl cells were treated with TNF-α (10 ng/mL) in the presence of the test compounds, and luciferase activity was measured at 24 h post-transfection. **(C)** Jurkat cells were co-transfected with pLTR-Luc or pLTRΔκB-Luc, pEF1-Tat and pRL-TK in the presence of the test compounds. Luciferase activity was measured 48 h post-transfection. **(D)** Jurkat cells were co-transfected with pGL3-LTR and pRL-TK in the presence of the test compounds. TNF-α (10 ng/mL) was added 36 h post-transfection. Luciferase activity was measured 48 h post-transfection. Pyrrolidine dithiocarbamate (PDTC) at 100 μM and flavopiridol at 20 nM were used as positive controls. Results were presented as the mean plus the standard deviations (*n* = 3).

Among the viral and cellular factors involved in HIV-1 gene transcription, the viral protein Tat and the NF-κB/Rel family of cellular transcription factors are the most important factors for LTR-mediated viral gene transcription. The promoter-proximal region of HIV-1 LTR contains two adjacent NF-κB binding sites. Upon a variety of stimuli (e.g. TNF-α) and cell activation, NF-κB can bind to LTR and activates HIV-1 transcription. To gain further insight into the mechanism of action of triptolide, this compound was examined for its direct effect on Tat and NF-κB-mediated HIV-1 gene transcription using reporter assays. As shown in Figure 4A and B, transfection with the Tat expression plasmid or treatment with 10 ng/mL TNF-α induced approximately 145- or 2.8-fold increase in luciferase activity in TZM-bl cells respectively. Under these conditions, triptolide reduced Tat-induced reporter activity in a dose-dependent fashion. Interestingly, triptolide appeared to slightly enhance TNF-α-induced luciferase production in TZM-bl cells.

In another system using Jurkat cells, the inhibitory effect of triptolide on Tat-induced gene expression was confirmed using a co-transfection experiment with an HIV-1 LTR-driven luciferase plasmid and the Tat expression plasmid (Figure 4C). Triptolide had no effect on TNF-α induced gene expression at the tested concentrations in Jurkat cells (Figure 4D). In addition, triptolide also reduced the Tat-mediated LTR activation in Jurkat cells transfected with the HIV-1 LTR containing two mutated NF-κB binding sites (Figure 4C), demonstrating that inhibition by triptolide is independent of the NF-κB signaling pathway. In both TZM-bl and Jurkat cells, triptolide did not affect basal LTR transcription activity at the tested concentrations (Data not shown). Collectively, these results suggest that triptolide inhibits Tat-mediated HIV-1 LTR-driven transcription.

### Triptolide specifically reduces steady-state level of Tat protein

**Figure 5:**
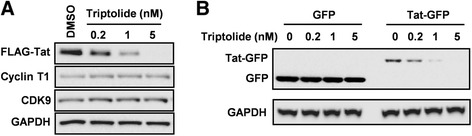
**Triptolide specifically reduces steady-state level of Tat protein. (A)** HeLa cells were transfected with pEF1-FLAG-Tat in the presence of increasing concentrations of triptolide. Cells were harvested 48 h post-transfection, and protein expression levels were determined by Western blotting. **(B)** HeLa cells were transfected either with pEF1-GFP (left) or pEF1-Tat-GFP (right) in the presence of increasing concentrations of triptolide. Cells were harvested 48 h post-transfection, and protein expression levels were determined by Western blotting.

In theory, triptolide may exert its activity through interfering with Tat protein expression or Tat function or both. Thus, we next investigated whether triptolide directly affects Tat protein expression level by transfecting HeLa cells with plasmid expressing FLAG-tagged Tat (the human elongation factor 1 α subunit promoter, EF1 α promoter). Strikingly, the steady-state level of Tat protein was reduced by triptolide in a dose-dependent manner as was determined with Western blotting (Figure 5A). Inhibition on Tat protein expression was a selective event and was not accompanied by the global suppression of cellular gene expression, since the expression levels of endogenous cyclin T1 and CDK9 remained unchanged in the presence of triptolide (Figure 5A). To exclude the possibility that triptolide acts against the transcription activity of EF1 α subunit promoter, we also constructed the plasmids expressing green fluorescent protein (GFP) and Tat-GFP fusion protein. The expression level of GFP was not reduced in the presence of triptolide (Figure 5B). However, this compound reduced the steady-state levels of Tat-GFP dose-dependently, indicating this compound does not affect the EF1α subunit promoter activity and specifically targets Tat protein. Taken together, these data strongly suggest that triptolide specifically reduces the steady-state level of Tat protein.

### Triptolide reduces Tat protein stability by enhancing proteasomal degradation of Tat

**Figure 6:**
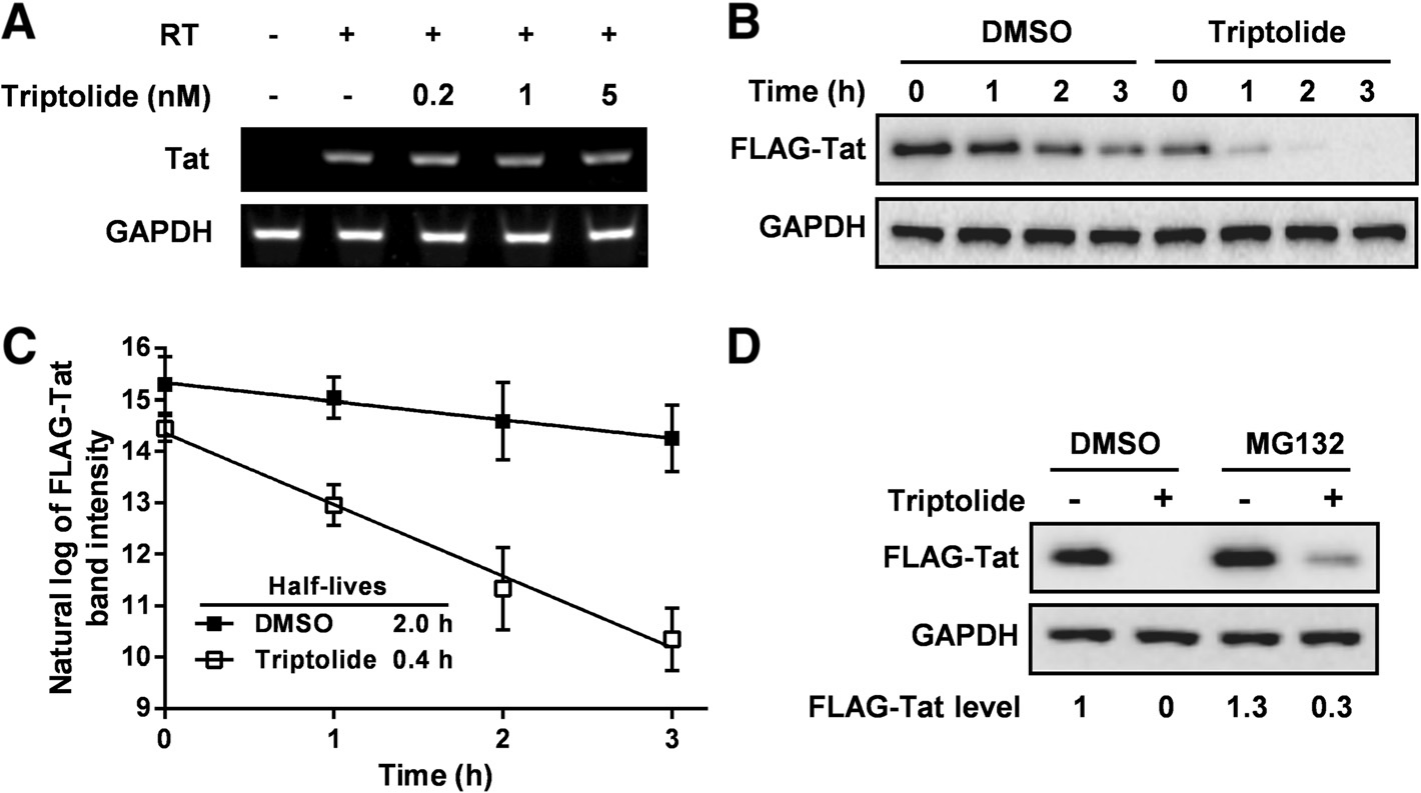
**Triptolide reduces Tat protein stability by enhancing proteasomal degradation of Tat. (A)** The effect of triptolide on Tat mRNA levels. HeLa cells were transfected with pEF1-Tat in the presence of increasing concentrations of triptolide. Cells were harvested 48 h post-transfection. The steady-state mRNA levels of Tat and GAPDH were examined using semi-quantitative reverse transcription PCR analysis. RT, reverse transcriptase. **(B)** The effect of triptolide on Tat protein degradation. HeLa cells were transfected with pEF1-FLAG-Tat in the presence of 5 nM triptolide or vehicle (DMSO). At 28 h post-transfection, cells were treated with 20 μg/mL of CHX to arrest protein synthesis. At 0, 1, 2, 3 h post-treatment, cells were harvested to determine protein expression levels using Western blot analysis. **(C)** The band intensities of FLAG-Tat in panel B were measured, and their natural log values were plotted as a function of time. Results were presented as the mean plus the standard deviations (*n* = 3). **(D)** Proteasome inhibition restores Tat protein expression. HeLa cells were transfected with pEF1-FLAG-Tat in the presence or absence of 5 nM triptolide. At 48 h post-transfection, cells were treated with 20 μM of MG132, and cultured for an additional 3 h before harvest. Protein expression levels were determined by Western blot analysis.

To investigate whether triptolide decreases Tat protein levels by reducing its mRNA stability, we transfected HeLa cells with the Tat expression plasmid and assessed the steady-state mRNA level of Tat in the presence or absence of triptolide using reverse-transcription PCR analysis. Notably, treatment with triptolide did not significantly alter Tat mRNA abundance (Figure 6A), indicating that this compound does not alter the stability of Tat mRNA but rather exerts its activity on later processes, such as mRNA translation or protein degradation.

Therefore, we next performed protein translation arrest experiments to investigate whether triptolide reduces Tat stability. HeLa cells expressing FLAG-Tat were pretreated with triptolide or DMSO for 28 h, cycloheximide (CHX) was then administrated. Cells were harvested in a time course. Western blotting results showed that FLAG-Tat was degraded more quickly upon the treatment of triptolide (Figure 6B). To calculate the half-lives of FLAG-Tat, the natural log values of band intensities were plotted against time (Figure 6C). The half-life of FLAG-Tat was determined to be 2.0 h, and it was reduced to 0.4 h in the presence of triptolide. Overall, these results showed that triptolide reduces Tat steady-state levels by decreasing Tat protein stability.

Tat degradation is regulated by proteasomal systems [25]. Theoretically, if triptolide enhances Tat protein degradation, inhibition of proteasomal degradation [26,27] could rescue the decreased Tat expression caused by triptolide. HeLa cells expressing FLAG-Tat in the presence or absence of triptolide were treated with the proteasomal inhibitor MG132 before probing with Western blotting. As shown in Figure 6D, MG132 treatment increased Tat level by 30%, indicating the amount of *de novo* synthesized Tat protein was approximately 30% of the content in DMSO control. In the presence of triptolide, Tat protein expression was reduced to undetectable level and restoration of Tat protein was observed after treatment with MG132 (also increased by 30% of Tat level in DMSO control). Thus, in the presence of MG132, the activity of triptolide on newly synthesized Tat protein was completely abolished. These data taken together suggest that triptolide specifically reduces Tat protein steady-state levels by enhancing proteasomal degradation of Tat protein.

### N-terminal domains of Tat are required for triptolide to reduce Tat steady-state levels

**Figure 7:**
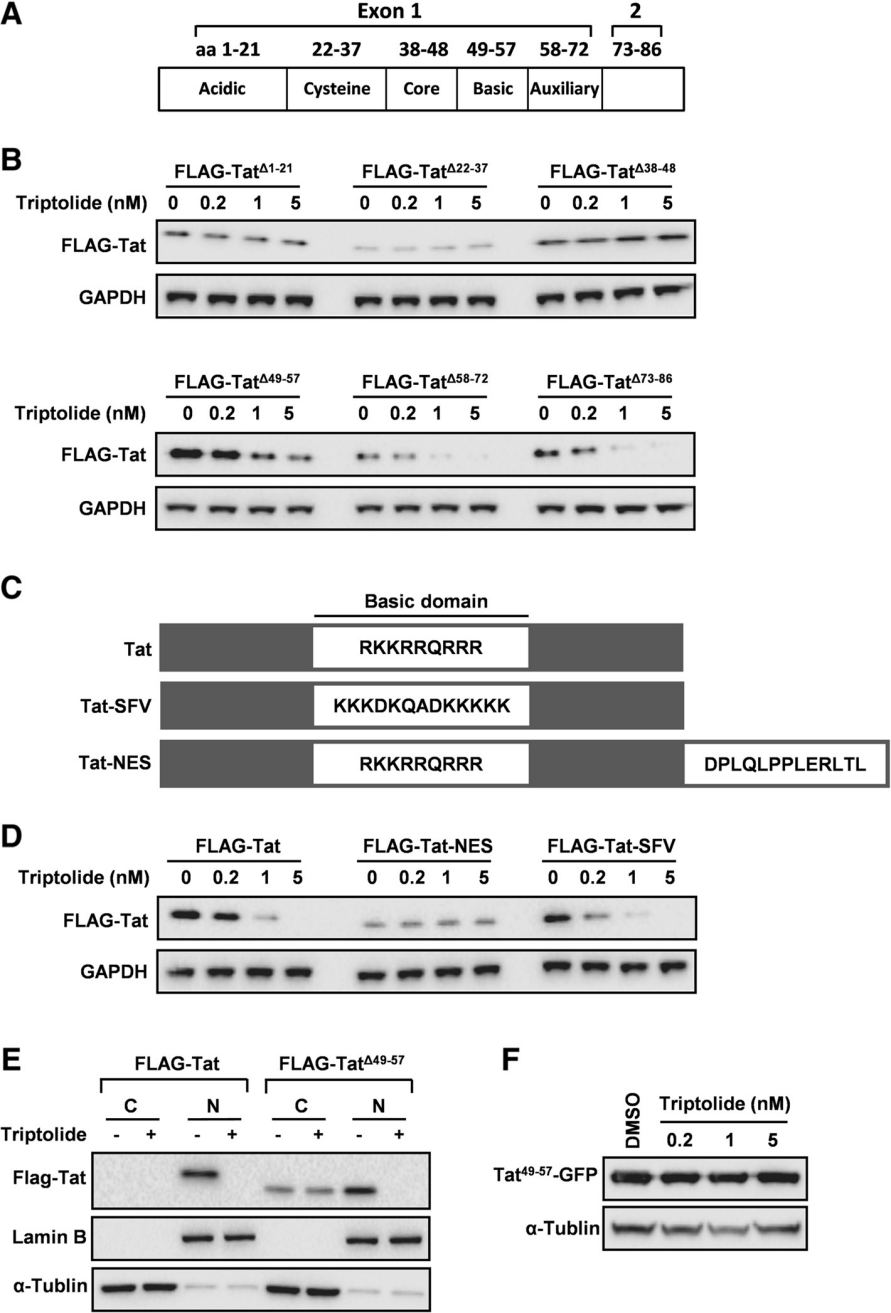
**N-terminal domains are required for triptolide to reduce Tat steady-state levels. (A)** Schematic diagram of domains of HIV-1 Tat. **(B)** The effects of triptolide on steady-state levels of Tat mutants. HeLa cells were transfected with plasmids encoding truncated forms of Tat in the presence of increasing concentrations of triptolide. Cells were harvested 48 h post-transfection, and protein expression levels were determined by Western blotting. **(C)** Schematic diagram of wild-type Tat and two mutants with domain substitution. In Tat-SFV, the basic domain was replaced with NLS from SFV capsid. In Tat-NES, the NES from HIV-1 Rev was fused to the C-terminus of Tat. **(D)** The effects of triptolide on steady-state levels of Tat mutants depicted in panel C. HeLa cells were transfected with plasmids encoding Tat mutants in the presence of increasing concentrations of tritpolide. Cells were harvested 48 h post-transfection, and protein expression levels were determined by Western blotting. **(E)** HeLa cells were transfected with pEF1-FLAG-Tat or pEF1-FLAG-Tat^Δ49–57^ in the presence or absence of 5 nM triptolide. At 48 h post transfection, the nucleus and cytoplasm were fractionated using NE-PER nuclear and cytoplasmic extraction reagent, and protein expression levels were determined by Western blotting. C, cytoplasm; N, nucleus. **(F)** HeLa cells were transfected with pEF1-Tat^49–57^-GFP in the presence of increasing concentrations of triptolide. Cells were harvested 48 h post-transfection, and protein expression levels were determined by Western blotting.

To further understand the mode-of-action of triptolide to induce Tat degradation, we tried to map the regions of Tat protein that confer the sensitivity to triptolide. Deletion mutants in six domains were generated in a plasmid expressing an N-terminal FLAG tag in a manner that encompassed the entire length of NL4-3 Tat protein (Figure 7A). HeLa cells were transfected with one of the domain deletion mutants, and the protein expression levels in the absence or presence of triptolide were assessed (Figure 7B). Results showed that the N-terminal regions, including acidic region, cysteine region and core region, are absolutely required, since the deletion of one of these regions completely aborted the activity of triptolide (Figure 7B). In contrast, the C-terminal regions (amino acid residues 58–86) are dispensable, and the potency of triptolide against these mutants was similar to that against wild-type Tat (Figure 7B). Interestingly, the effect of triptolide was partially attenuated with the deletion of basic region, indicating that basic region is also involved in the mode-of-action of triptolide (Figure 7B).

Tat basic domain contains two arginine methylation sites and a nuclear localization signal (NLS). Previous studies showed that arginine methylation in the basic domain increases the stability of Tat, and the posttranslational modification occurs in the cell nucleus [28]. So we generated two FLAG-Tat mutants to study the involvement of arginine methylation and nuclear localization in triptolide’s mode-of-action. In the first mutant (FLAG-Tat-SFV), the basic domain was replaced with the NLS from Semliki Forest virus (SFV) capsid protein (without arginine residues, Figure 7C) [29]. In the other mutant, the basic domain was kept intact and a nuclear export signal (NES) from HIV-1 Rev [30] was fused to the C-terminus of Tat (Figure 7C). HeLa cells were transfected with either wild-type FLAG-Tat, or one of the mutants in the presence of different concentrations of triptolide to assess the effect of these mutations on triptolide mediated degradation of Tat. Strikingly, the steady levels of FLAG-Tat-NES remained unchanged despite the presence of an intact basic region, indicating nuclear localization of Tat is critical (Figure 7D). In sharp contrast to FLAG-Tat-NES, FLAG-Tat-SFV expression was dose-dependently reduced by triptolide (Figure 7D), suggesting arginine methylation is not required. Based on these data, we conclude that nuclear localization of Tat, but not arginine methylation, is required for triptolide to reduce Tat steady-state levels.

To further confirm that nuclear localization is required for triptolide’s action, the nuclear/cytoplasmic protein separation assay was performed. As shown is Figure 7E, the wild-type Tat protein predominantly localized to the nucleus. With basic domain deleted, Tat protein (Tat^Δ49–57^) could localize to both cytoplasm and nucleus (Figure 7E). In such conditions, triptolide only acted on Tat protein accumulated in nucleus, but not Tat protein accumulated in cytoplasm (Figure 7E). These observations explained why triptolide was partially active against basic domain-deleted Tat mutant (Figure 7B). Next, we tested triptolide’s activity on the fusion protein of Tat basic domain with GFP (Tat^49–57^-GFP). The results indicated that basic domain alone was not sufficient to mediate the susceptibility to triptolide (Figure 7F). Taken together, N-terminal domains of Tat (amino acid residues 1–57) are required for triptolide to reduce Tat steady-state levels.

## Discussion

Although the advent of HAART changed the landscape of HIV/AIDS, there is still no cure for this disease currently available. Therefore, the discovery and development of novel therapeutics for HIV infection is still necessary. In this study, we found that triptolide inhibited HIV-1 replication by prompting Tat protein degradation, a novel mechanism of action. This compound was identified as a novel HIV-1 replication inhibitor in a cell-based assay using TZM-bl cells. Other *in vitro* antiviral assays demonstrated that this compound also specifically inhibits HIV-1 replication in the Jurkat T cell line and in PBMCs (Figure 2). After the validation of antiviral activity, we performed time-of-addition experiments and PCR analysis to determine the step(s) affected by this compound. The results of these experiments demonstrated that triptolide acts at a post-integration step and blocks viral gene transcription (Figure 3). Further study showed that triptolide can reduce Tat-induced LTR activation, confirming the conclusion that this compound targets viral gene transcription (Figure 4). Interestingly, treatment with triptolide led to a reduction of steady-state levels of Tat protein in transient transfection systems. The suppression of Tat expression was specific, because the expression of GFP using the same vector was not affected. More importantly, the expression levels of tested cellular genes, which are essential for LTR transcription activity, were not impaired (Figure 5). PCR analysis of cells transfected with the Tat expressing plasmid revealed that Tat mRNA level remained unchanged upon treatment. However, proteasomal inhibition rescued down-regulated Tat protein expression, indicating that triptolide acts at the stage of Tat protein degradation (Figure 6). These data taken together suggest that triptolide interferes with Tat protein degradation and thus blocks acute HIV-1 infection at the stage of viral transcription. Considering that Tat-mediated viral transcription is a common event in all forms of HIV-1 replication, triptolide is also expected to inhibit virus production from chronically and latently infected cells. Further studies should be performed to determine whether triptolide also provides therapeutic benefits in chronic and latent infection systems.

Control of HIV-1 gene transcription is an attractive approach for anti-HIV-1 chemotherapy. However, attempts to repress viral transcription present several unique challenges, because most factors involved in HIV-1 transcription are host general transcriptional regulators and cofactors, that are required for host gene expression. For example, although compounds targeting P-TEFb and other factors have been shown to block HIV gene expression, these compounds have a global impact on cellular gene expression and thus lack specificity. Therefore, the clinical potential of compounds that targeting the host transcription machinery is very limited due to safety concerns.

Although triptolide has been reported to possess multiple bioactivities that interfere with host transcriptional factors at higher concentrations, none of the activities were responsible for the unique antiviral profile observed in this study. In previous studies, triptolide was found to interfere with TNF-α-induced NF-κB activation [31]. The cellular factor NF-κB is a potent activator of HIV-1gene transcription, and many NF-κB inhibitors have been reported to block viral gene expression and replication [32]. However, the highest concentration we used in our assays was only 5 nM, which was not active against TNF-α-induced LTR activation in our reporter assay (Figure 4B and D). Furthermore, triptolide showed comparable potency against Tat-induced LTR and LTRΔκB activation (Figure 4C). Taken together, these results showed that the antiviral activity of triptolide could not be attributed to NF-κB inhibition. In other reports, triptolide was shown to inhibit global gene transcription by inducing the degradation of RNA polymerase II [33], and inhibiting the ATPase activity of XPB, a subunit of the general transcription factor TFIIH [34]. These results may raise the concern that triptolide blocks Tat protein expression by nonspecific disruption of the cellular transcription machinery. However, several lines of evidence should relieve this concern. First, this compound possessed a therapeutic window in all cell-based assays tested. Second, the expression levels of exogenous gene (GFP, in the same vector) and several endogenous genes tested are not affected by triptolide in the transient transfection assay (Figure 5A and B). Third, the basal transcriptional activity of HIV-1 LTR, which is regulated by a large number of host transcriptional activators [35,36], remained unchanged in the presence of triptolide (Data not shown). Thus, these results indicated that triptolide specifically targets Tat protein, without effect on global cellular gene expression.

To further understand the mechanism, we also performed mutation studies and identified N-terminal domains of Tat and nuclear localization are essential for the action of triptolide (Figure 7). Although the molecular details of Tat degradation caused by triptolide remain unclear, the specific and unique mode of action warrants further investigation. Posttranslational modification of Tat protein was reported to regulate Tat protein stability [29]. Although we have demonstrated that arginine methylation in basic region is not required for triptolide to prompt Tat degradation, we cannot rule out the possibility that triptolide may interfere with other forms of post-translational modification in its N-terminal domains, and thus reduces Tat stability. Other reports indicated that interaction between Tat and its partners can stabilize Tat expression levels [37,38]. This raises an alternative possibility that triptolide disrupts the interaction between Tat and its stabilizers and sensitize Tat to degradation. Further studies should be performed to determine the exact mechanism based on these hypotheses.

As mentioned above, several previous studies have indicated that triptolide may interfere with important cellular functions at higher doses [31,33,34,39-41]. This property may be the reason why triptolide exhibited a limited therapeutic window (SI = 7.5 ~ 79.4, depending on the cell type and virus strain) in our *in vitro* antiviral assays. Thus, despite the novel mode of action, the clinical potential of triptolide in its current form may still be limited due to potential safety concerns. In this regard, synthetic chemistry studies should be performed to explore the structure activity relationship (SAR) of triptolide, define the chemical groups responsible for its antiviral activity, and test whether other unwanted bioactivities could be eliminated through chemical modification. Potentially, potent derivatives of triptolide that avoid undesired activities may be developed. Of course, it is also a possible that the chemical nature of triptolide makes it unsuitable for further drug development.

## Conclusion

Our results presented here demonstrate that triptolide inhibits HIV-1gene transcription and replication by prompting proteasomal degradation of Tat protein, a novel anti-HIV-1 mechanism. Although the therapeutic utility of triptolide in its current form may be limited due to safety concerns, triptolide may serve as a starting point for developing novel HIV-1 therapeutic approaches or as a basic research tool for interrogating events during viral replication.

## Methods

### Cell culture, plasmids and viruses

HeLa, 293 T and Jurkat cells were obtained from American Type Culture Collection (ATCC). TZM-bl cells (catalog no.8129) and MT-4 cells (catalog no.120) were obtained through the NIH AIDS Research and Reference Reagent Program (National Institute of Allergy and Infectious Diseases). PBMCs were isolated from healthy donors by using Ficoll-Paque PREMIUM (GE Healthcare) according to the manufacturer’s instructions. HeLa and TZM-bl cells were propagated in Dulbecco’s modified Eagle’s medium (DMEM) (Invitrogen) supplemented with 10% FBS (Gibco), 100U/mL of penicillin and 0.1 mg/mL of streptomycin. Jurkat cells, MT-4 cells and PBMCs were grown in RPMI 1640 supplemented with 10% FBS and 10 μg/mL of gentamicin.

The HIV-1 molecular clone pNL4-3 (catalog no.114), pLAI.2 (catalog no.2532) and the env-deficient proviral clone pNL4-3.Luc.R-E- (catalog no.3418) were obtained through the AIDS Research and Reference Reagent Program. HIV-1 Tat expression plasmid pEF1-Tat, pEF1-FLAG-Tat were generated by cloning the full-length Tat coding sequence (containing both exon 1 and exon 2) of HIV-1 NL4-3 or FLAG-Tat into pEF1-V5/His-A (Invitrogen), respectively. FLAG-Tat mutants expressing plasmids were constructed by standard molecular cloning methods. pGL3-Basic and pRT-TK were purchased from Promega. pLTR-Luc was constructed by inserting the HIV-1 NL4-3 5′ LTR region (1 ~ 789) into pGL3-Basic. pLTRΔκB-Luc was constructed by deleting two NF-κB binding sites from pLTR-Luc.

The HIV-1 strains NL4-3 and LAI were generated by transfecting the corresponding plasmids into 293 T cells and propagating them in MT-4 cells. The HIV-1 strain BaL was kindly provided by Dr. Qinxue Hu (Wuhan Institutes of Virology, Chinese Academy of Sciences). The single-round reporter virus HIV-1-Luc was generated by co-transfecting 293 T cells with pNL4.3R-E-Luc and the plasmid expressing the HIV-1 NL4-3 envelope protein.

### Antibodies and compounds

The anti-FLAG antibody (F1804) was purchased from Sigma-Aldrich. Antibodies to Glyceraldehyde-3-phosphate dehydrogenase (GAPDH) (#2118), α-Tublin (#3873), GFP (#2555), cyclin T1 (#8744) and CDK9 (#2316) were purchased from Cell Signaling Technology. The anti-Lamin B antibody (SC-374015) was purchased from Santa Cruz Biotechnology. All horseradish peroxidase labeled secondary antibodies were obtained from Thermo Scientific.

The compounds isolated from Chinese traditional herbs for the anti-HIV-1 drug screen were purchased from Sichuan Weikeqi Biological Technology Co., Ltd., China. The purity of the compound was ≥ 98%, as was determined by high-performance liquid chromatography analysis. The compound samples were solved in dimethyl sulfoxide (DMSO) at a concentration of 50 mM. The following reference compounds were obtained through the NIH AIDS Research and Reference Reagent Program, Division of AIDS, NIAID, NIH: zidovudine (catalog no. 3485), integrase inhibitor 118-D-24 (catalog no. 9957) and (−) flavopiridol (catalog no. 9925). Dextran sulfate sodium salt (average molecule weight of 5,000 Da), PDTC, MG132 and CHX were purchased from Sigma-Aldrich.

### In vitro anti-HIV-1 assays

The anti-HIV-1 activities of compounds were evaluated in a variety of assays, including the TZM-bl assay, reporter virus assay, and PBMCs-based antiviral assay. In the TZM-bl assay, TZM-bl cells were plated at a density of 1 × 10^4^ cells per well in 96-well tissue culture plates one day before infection. On the day of the experiment, the cell supernatant was removed, and 100 μL of each serially diluted compounds was added were added. HIV-1 NL4-3 in 50 μL of complete medium was then added to each well to achieve a multiplicity of infection (MOI) of 0.5. At 48 h post-infection, the luciferase activity in the cells was analyzed with the SteadyGlo reagent (Promega) according to manufacturer’s instructions. The luminescent signal was measured using the EnVison 2102 Multilabel Reader (Perkin Elmer). The EC_50_ (50% effective concentration) values correspond to compound concentrations that resulted in a 50% reduction in luciferase activity.

In the antiviral assay with the HIV-1 reporter virus, Jurkat cells were seeded at a density of 2 × 10^4^ cells per well in 96-well tissue culture plates and infected with HIV-1-Luc at an MOI of 0.5 in the presence or absence of the test compounds. At 48 h post-infection, luciferase activity in the cells was determined and EC_50_ values were calculated as described above.

In the antiviral assay using PBMCs, determination of the anti-HIV-1 activity of a particular compound was based on the inhibition of HIV-1 capsid protein (p24) production. After isolation, PBMCs were stimulated with phytohemagglutinin (2 μg/ml, Sigma-Aldrich) and interleukin-2 (20 U/mL, Sigma-Aldrich) in RPMI 1640 complete medium for three days and were then maintained with interleukin-2 during viral infection. PBMCs were infected with HIV-1 NL4-3 (MOI = 0.01). After viral adsorption for 2 h, the cells were washed thoroughly with culture medium to remove unabsorbed viral particles. The infected cells (2 × 10^5^ cells/well) were cultured in 96-well plates in the presence of or absence of the test compound. At two, four, six, and eight days post-infection, half of the culture supernatant was collected and replaced with fresh medium containing the test compounds. The level of viral replication was determined with an HIV-1 p24 antigen capture enzyme-linked immunosorbent assay (ELISA). The EC_50_ values corresponded to compound concentrations that resulted in a 50% reduction in p24 production at day 6 post-infection.

The cytotoxicity of the tested compounds was evaluated in parallel with their antiviral assays using the CellTiter-Glo reagent (Promega) according to the manufacturer’s protocol. The 50% cytotoxic concentrations (CC_50_s) is the compound concentration that reduced cell viability by 50%.

### Time-of-addition experiment

TZM-bl cells seeded in 96-well plates (2 × 10^4^ /well) were infected with HIV-1 NL4-3 (MOI = 1). The virus inoculum was removed after 1 h of infection. Test compounds were added at different time points after infection (0 h, 2 h, 4 h, 6 h, 8 h, and 10 h). Dextran sulfate was added at 10 μg/mL, zidovudine at 1 μM and triptolide at 5 nM. At 48 h post-infection, the virus replication level was determined by analyzing luciferase activity in the cells as described above.

### Semi-quantitative PCR analysis

The effects of the test compounds on HIV-1 proviral DNA and mRNA synthesis were analyzed by PCR. Jurkat cells were grown in 24-well plates and infected with the HIV-1 NL4-3 strain at an MOI of 0.5, and then test compounds were added at the desired concentrations. After incubation for 12 h, genomic DNA and mRNA from the infected cells were isolated using a Genomic DNA Mini Preparation Kit (Beyotime, China) and TRIzol reagent (Invitrogen Life Technologies), respectively. Nested PCR was used for the amplification of integrated proviral DNA, as previously described [42]. To determine viral mRNA expression levels, 1 μg of RNA was treated with RQ1 RNase-Free DNase (Promega) and reverse transcribed using M-MLV Reverse Transcriptase (Promega) and random primers (Promega). An aliquot of cDNA was used as template for amplification of the HIV-1 Gag region as described elsewhere [43]. As DNA and RNA input controls, genomic DNA and cDNA was subjected to GAPDH amplification using the primers 5′-GAAGGTGAAGGTCGGAGTC-3′ and 5′-GAAGATGGTGATGGGATTTC-3′.

PCR analysis was also used to examine the effect of a particular compound on Tat mRNA expression. Sub-confluent HeLa cells were grown in 24-well plates and transfected with pEF1-Tat in the absence or the presence of the test compounds. Total mRNA was extracted at 48 h post-transfection, and reverse transcription was performed as described above. Then, the cDNA was subjected to Tat gene amplification using the primers 5′- GAAGCATCCAGGAAGTCAGCC -3′ and 5′-ACAAACTTGGCAATGAAAGCAACAC-3′. GAPDH was used as an input control.

All of the PCR products were visualized on a UV transilluminator in 2% agarose gels stained with ethidium bromide. In preliminary experiments, the exponential range of the PCR amplification curve was determined for all of the PCR products by varying the amount of input DNA and the number of PCR cycles. Based on these experiments, appropriate conditions were chosen to perform semi-quantitative PCRs.

### HIV-1 LTR-luciferase reporter assay

The effect of the test compounds on HIV-1 LTR promoter activity was evaluated using the luciferase reporter systems in both TZM-bl and Jurkat cells. TZM-bl cells seeded in 96-well plates (2 × 10^4^ cells/well) were treated with either 10 ng/mL TNF-α (Sigma-Aldrich) or co-transfected with pEF1-Tat and pRL-TK (for transfection efficiency normalization) using Lipofectamine 2000 (Invitrogen). The cells were then cultured in the presence of various concentrations of the test compounds. After 24 h (TNF-α stimulation) or 48 h (Tat transfection) of incubation, luciferase activity was determined from cell lysates using the StedyGlo reagent (for TNF-α treated cells) or a dual luciferase assay system (Promega, for transfected cells) as recommended by the manufacturer.

Jurkat cells were seeded in 24-well plates at a density of 2 × 10^5^ cells per well. For TNF-α induction, cells were co-transfected with pLTR-Luc and pRL-TK in the presence or absence of the test compounds. Then, the cells were treated with 10 ng/mL TNF-α 36 h post-transfection. After induction for an additional 12 h, luciferase activity was analyzed. For Tat-induced LTR activation, Jurkat cells were co-transfected with pLTR-Luc (or pLTR-Luc-ΔkB), pEF1-Tat and pRL-TK, and cultured in the presence of various concentrations of test compounds for an additional 48 h before the determination of luciferase activity. Luciferase levels were expressed relative to levels in cells without induction.

### Western blot analysis

Cells in 12-well plates were transfected using Lipofectamine 2000 and treated as indicated in figure legends. Cells were washed with phosphate buffered saline (PBS) and lysed with radio immunoprecipitation assay lysis buffer supplemented with complete mini protease inhibitor cocktail (Roche) at 4°C for 1 h. Proteins from cell lysates were separated by SDS-PAGE and transferred to polyvinylidene difluoride membranes. After overnight incubation with primary antibodies at 4°C, each blot was probed with horseradish peroxidase-conjugated secondary antibody. Immunoreactive signals were detected with an enhanced chemiluminescence substrate (Thermo Scientific) using an AlphaEaseH FC Imaging System (Alpha Innotech Corporation).

### Protein translation arrest

HeLa cells were transfected with pEF1-FLAG-Tat in the presence of 5 nM triptolide or vehicle (DMSO). After 28 h, cells were treated with 20 μg/mL CHX to arrest protein synthesis. Cells were harvested at 0, 1, 2, 3 h post-CHX treatment and Western blotting was performed. FLAG-Tat half-lives were determined as previously described [28].
